# Supplementary material for: Are carbon emissions trading and green financial instruments synergistic? -Comprehensive quantitative research based on content analysis
Source: PLoS One. 2024 Mar 7;19(3):e0298601. doi: 10.1371/journal.pone.0298601 (PMC10919601; doi:10.1371/journal.pone.0298601)
Supplement: S1 Data — (ZIP) [file pone.0298601.s001.zip › Data/地级市Z_F工作报告.zip!/░▓╗╒/║╧╖╩╩╨/└┤╫╘╣1⁄2╓┌║┼í╛┬φ┐╦╩2╛▌═°í┐_2007.docx]

# **2007年政府工作报告**

发布时间：2007-01-31 17:07

【字体：[大](http://www.hefei.gov.cn/public/1741/javascript:void(0);) [中](http://www.hefei.gov.cn/public/1741/javascript:void(0);) [小](http://www.hefei.gov.cn/public/1741/javascript:void(0);)】

各位代表：

现在，我代表市人民政府，向大会作政府工作报告，请予审议，并请市政协委员和其他列席人员提出意见。

一、２００６年政府工作回顾

２００６年是“十一五”开局之年，全市人民全面贯彻落实科学发展观，紧扣“大发展、大建设、大环境”这个主题，着力创新发展模式，全市综合经济实力大幅提升，县域经济取得重要突破，基础设施建设进一步加快，社会各项事业全面发展，投资发展环境明显改善，人民群众得到更多实惠，经济社会呈现出又好又快的发展态势。地区生产总值１０７３．８６亿元，提前４年实现ＧＤＰ千亿元发展规划，增长１７．５％，为１９９７年以来最高增幅，增速在全国省会城市中名列前茅。省会经济首位度达１７．５％，比上年提高１．２个百分点；实现财政收入１６７．７７亿元，增长２８．２％，总量占全省２０．６％，其中地方财政收入７９．４亿元，增长３７．８％。国税收入突破１００亿元，地方税收突破５０亿元。全社会固定资产投资８２４．８亿元，占全省２３．３％，增长６６．５％，创１９９３年以来最高增幅，增速位于全国省会城市前列。社会消费品零售总额３８４．３亿元，增长１８．５％。城镇居民人均可支配收入１１０１３元，增长１３．７％；农民人均纯收入３６９０元，首次超过全国平均水平，增长１５．１％。城镇登记失业率控制在４．３％以内，人口出生率控制在９．８１‰。２００６年中国城市竞争力研究报告显示，合肥的城市整体竞争力由上年的第３１位上升到第２９位，城市成长竞争力从第３２名前移到第２９名。

过去一年，主要抓了８个方面工作：

（一）强化“工业立市”战略，整体提升经济发展的实力和水平。组织实施“加快工业发展年”活动，出台《加快新型工业化发展若干政策》、《免收开发园区工业投资项目行政事业性收费》等政策文件，设立工业发展专项资金，推进“１２１”工程，工业经济呈现出投入大、增长快、效益好的崭新局面。全年工业投资１９３．４亿元，比上年增长１．１６倍。新增规模以上企业１８３户，规模以上工业总产值１０９８．５亿元，提前２年实现千亿元规划目标。工业运行水平显著提高，规模以上工业实现增加值３６３．２亿元，比上年增长２１．７％；实现利润增长４７．９％，高于上年１６．８个百分点；亏损企业减亏５１．５％，减亏幅度居全省第一；工业经济效益综合指数达到２０９％，位列全省第一，高于全省平均水平３０多个百分点。建筑业持续快速发展，全年完成产值４４５．６亿元，增长４３．９％。制定鼓励重点服务业发展政策，促进第三产业优化结构、提高水平。加快实施“双百市场”和“万村千乡”市场工程，城乡市场体系不断完善。打造会展品牌，成功举办全国农机展、全国苗木花卉展、首届中国徽菜美食节和首届中国坚果炒货食品节等大型展会。加大旅游宣传和促销力度，全年实现旅游总收入６８．４亿元，增长１９．５％。树立“发展为上，投资为本”的理念，千方百计加大投入，固定资产投资高速增长，投资结构进一步优化，企业自主投资能力明显增强。重点项目进展顺利，“１３４６”行动计划项目共完成投资３２１．１亿元，其中，列入“８６１”计划项目完成投资１３７．４亿元。（

（二）加快发展县域经济，扎实推进社会主义新农村建设。紧紧围绕提高农民收入这个核心，全力推进“县域突破”战略，县域经济呈现出跨越式发展态势。３县地区生产总值、财政收入、农村固定资产投资均高于全市平均增长水平。强化“工业立县”措施已见实效，３县规模以上工业实现增加值７１．１亿元，比上年增长２７．３％，肥西县成为全省首个规模以上工业产值超百亿县。大力发展农业产业化，各类农副产品加工企业发展到３２６家，市级以上龙头企业７２家，伊利乳业等大型龙头企业建成投产。农业结构调整步伐加快，菜瓜果规模、档次大幅提升，规模养殖比重达到６５％以上。粮油生产能力稳步提高，苗木花卉基地不断扩大，休闲观光农业加快发展。农业标准化、品牌化工程扎实推进，全市已获认证的无公害农产品达８１个、绿色食品５７个、省名牌农产品３１个，分别新增３６个、４４个和１０个。“阳光工程”取得积极成效，全年培训农民１．４万人，转移就业率８０％以上。社会主义新农村建设取得积极进展。出台《“十镇百村”示范工程实施方案》，完成首批１０个示范镇、６７个示范村规划并开始实施，一批先行示范点建设成为全省新农村建设的典型。大力实施防洪保安、农田水利和农村饮水安全工程，改善农村水利设施条件。加快农村道路建设，５０％以上行政村实现“村村通水泥路”，通达里程１１００公里。推进农村宅基地整理，村容村貌有所改善。

（三）认真落实“１４１”城市空间发展战略，拉开滨湖大城市建设序幕。把握合肥城市从“环城”走向“滨湖”、“临江”的历史性趋势，开展新一轮城市总体规划、近期建设规划和土地利用总体规划编制工作，统筹推进“１４１”城市空间发展战略。通过国际招标，高水平完成滨湖新区规划，编制完成城市交通、基础设施等专业规划。启动滨湖新区建设，起步区道路及社会事业等配套设施建设进展较快。省会交通枢纽建设取得新进展，合六叶、合淮阜合肥段，环城高速建设及合宁、合武铁路建设进展顺利，新机场及集装箱港口前期工作基本完成。中心城区到四大组团及滨湖新区快速通道建设全面展开。开工建设金寨路高架桥及主城区３０多条道路改扩建工程，其中７条道路已建成通车。加快发展供电、供水、供气、供热及污水处理、市容环卫、公共交通等公用事业，不断完善城市服务功能。建立查处违法建设长效工作机制，巩固和扩大拆违成果。结合大拆违，改造了一批小街巷，打通了一批“断头路”。加快城市园林绿化建设，实施高压走廊一期、三国遗址公园等重点项目工程，新建各类园林绿地８３００亩。对重点污染源实施综合整治，继续创建国家环保模范城。规范房地产市场秩序，房地产市场呈现健康稳步发展态势。实施合瓦路等危改项目２５个，完成整治旧小区８个。

（四）加快科技创新型试点市建设，培育城市核心竞争力。围绕把科教优势转化为产业优势，进一步完善发展规划，组织实施以企业技术创新、体制机制创新、创新创业服务工程为主要内容的综合创新工程，全力以赴推进科技创新型试点市建设。设立试点市工作专门办事机构，分解落实试点工作任务，出台一系列支持政策。设立并发挥１亿元创新基金的引导作用，重点扶持２０家初具规模的高新技术企业。组织实施科技创新型企业培育计划和科技小巨人计划，在３５家企业开展试点，１２０家企业进入培育计划。全年高新技术产业实现总产值６３１亿元，技工贸总收入５９３．７亿元，分别增长２９．３％和２８．４％，新认定高新技术企业、高新技术产品分别达到５４家和５０个。积极构建以企业为主体的技术创新体系，新建国家级企业技术中心１家、省级工程研究中心１家和工程技术研究中心４家，新增国家级科技企业孵化器１家，合肥民营科技园创业中心被认定为国家级高新技术创业服务中心。建成制造业信息化生产力促进中心，合肥国家知识产权试点城市通过验收。加快试点市示范区建设，共完成投资近１０亿元，安徽循环经济技术工程研究院等１０个项目开工建设或建成投入使用；机电产业园开工建设，通用环境公司等１０余家企业和合肥微软技术中心等一批国内外知名大企业研发中心相继进驻示范区。引进风险投资，扩大担保范围，筹建了合肥科技农村商业银行。成功举办第六届中国•合肥高新技术项目－资本对接会，国家级金融机构首次冠名支持，参展参会近１０万人次，签约项目２５个，总投资５７亿元，协议资金５２亿元；银企对接项目２０多个，贷款额２０多亿元。

（五）加大招商引资力度，全方位扩大对外开放。加快实施东向发展战略，加强与国内外其他地区的交流合作，形成全方位扩大对外开放格局。积极参加徽商大会等境内外重点招商活动，组建１３０６支招商小组驻外招商，掀起招商引资新高潮。全年招商引资总量３３２．８亿元，增长６２．９％；引进内资项目１９２６个，引进市外资金２７５亿元，分别增长２２．３％和６０．２％；新批外商投资企业１５５户，实际利用外资７．２２亿美元，分别增长７０．３％、７７．６％。引资结构明显改善，工业实际引资１４３．７亿元，占全市招商引资总量４３．２％；新批千万美元以上外资项目４３个，增长４３７％，日本三井物流、英国翠丰、韩国鲜京等世界５００强企业在我市投资落户。招商领域进一步拓宽，千禧鹤、雨润、和威等涉农大企业集团落户合肥，美国沃尔玛、德国麦德龙、法国欧尚等世界５００强商业企业成功签约。加快开发区建设和发展，３大开发区完成投资和工业增加值分别增长４８．２％、２６．２％，占全市的２４．１％、４２．８％。优化对外贸易结构，鼓励企业参与国际竞争，积极开展对外经济技术合作，进出口贸易总额在中部城市继续位居前列。全年海关进出口总额４８．７６亿美元，增长１６．６％，其中出口３４．０７亿美元，增长２２％。完成外经营业额３．４亿美元，占全省８１％。外事、侨务工作取得新进展，对外交往的广度和深度进一步增强。

（六）推进体制机制创新和“效能革命”，大力优化发展环境。强力推进国有企业改革，合钢改革重组后，迅速焕发生机和活力，由重组前月亏损３０００多万元转变为月赢利８００万元。流通企业、公用事业企业改革加快推进，中小企业“双退”工作基本完成。加大企业上市和直接融资指导工作力度，推进上市公司股权分置改革。深入推进国有资产管理体制改革，党政机关与兴办经济实体脱钩改制、经营性事业单位改制转企工作基本结束。加快投融资体制改革，进一步整合资源，建立统一的融资平台和管理中心，全年融资５５亿元，是上年的２．８倍。改革城市规划管理体制和运行机制，积极探索建立运作有序、管理规范、服务高效的城乡规划管理体系。进一步完善土地管理制度，强化节约集约用地措施，建立全市统一经营性用地管理办法，清理闲置土地，提高土地利用率，土地市场秩序进一步规范。创新工程建设管理模式，建立工程建设管理责任体系，全市大建设项目实行有效最低价中标制度，对所有拆迁安置实行“三榜公示”，最大限度地保护群众的合法权益。建立全市工程建设、土地拍卖、产权交易、政府采购统一招投标市场，净化了招投标市场环境，节约了财政性资金。积极推进政府非税收入管理改革，全面清理撤销市直单位银行账户，实行预算内、外资金统一使用和管理。深入推进城市管理相对集中行政处罚权工作，健全以责任制和责任追究制为核心的长效管理机制。大力推进电子政务，全市统一的电子政务信息处理平台开始运行，城市级电子政务体系初步形成。

深入开展“机关效能建设年”活动，大力推进机关“效能革命”。深化行政审批制度改革，市级行政许可事项由３５３项减至２３０项；全面推行行政执法责任制，市直单位向全社会公开承诺办事范围、程序和效率。强化制度建设，严格实行“四条禁令”及“三项制度”，对部门审批实行“超时默认制”，对联合审批实行“缺席默认制”，政风有了明显好转。继续推行经济责任审计，全年对５２名党政领导干部和企业领导人员进行了经济责任审计。加强廉政建设，查处了一批违法违纪案件。制度创新和效能革命大大改善了投资发展环境，我市成为全国投资环境５０优城市、跨国公司眼中最具投资价值及投资潜力的中国城市、浙商（省外）最佳投资城市。

（七）加强精神文明建设，全面发展各项社会事业。以创建全国文明城市为统揽，深入开展“八荣八耻”教育，广泛开展各种群众性精神文明创建活动。庐州讲坛、科学家企业家讲坛等学习型城市建设活动有声有色。大张旗鼓地整治“四乱”，集中开展户外广告、店招标牌、摊群点、“牛皮癣”等专项整治，环境卫生和市容市貌显著改善。着眼于更好地满足人民群众的需求，协调发展社会事业。坚持教育是民生之基，始终把教育放在优先位置。完成新一轮教育发展规划，深化教育改革，促进教育公平与均衡发展。取消“同城借读”，规范教育收费，扩大省级示范高中招生规模，严禁公办学校义务教育阶段跨区招生。全年资助市区低保家庭学生和贫困生近万人，２１１７名农民工子女免收借读费。强力推进住宅小区配套３３所中小学建设，新建、扩建教学用房４．２４万平方米，开工建设全省第一流滨湖高中、初中学校，基本消除农村学校原有Ｄ类危房，新增民办教育机构４１所，有效缓解了就学压力。完善公共卫生服务体系，建成市、县疾控中心和市传染病医院、妇幼保健中心，预防保健和疾病控制水平进一步提高。新增社区卫生服务机构３５个，社区卫生服务网络基本建成，人口覆盖率达到８５％。新型农村合作医疗制度覆盖３县所有乡镇，改扩建乡镇卫生院近６０所，农村卫生综合服务能力不断提高。启动惠民医疗工程，医疗救助制度进一步健全。完善医疗服务体制改革，在全市２６家二级以上医院实行医疗检查、医学影像检查部分项目互认制度，市属医院试行３５个单病种限价收费制，规范医疗机构药品集中招标采购。继续推进文化强市战略。市体育中心建成投入使用，广电中心、文化艺术中心建设进展顺利。加快有线电视网络建设，启动数字电视工程。创作编排了多场文艺演出和大型音舞诗画《玉兰赋》，成功举办了首届国际友城艺术节、第二届中国书法兰亭奖和合肥工笔重彩画晋京展。庐剧入选首批国家非物质文化遗产保护名录。整顿和规范文化经营秩序，开展了网吧集中整治。农村实现村村通广播和电视全覆盖。新一轮市志修编工作进展顺利。成功申办第４届全国体育大会，在省第十一届运动会上获金牌、奖牌、总分和体育道德风尚奖４个第一。人口增长保持低生育水平，出生人口性别比综合治理工作扎实开展。国防后备力量建设不断加强，双拥、人防工作持续进步。科普和学术活动亮点纷呈，防震减灾综合防御能力显著提高，保密、对台、民族、宗教工作取得新进展，气象、统计、档案、妇女儿童、未成年人保护、残疾人、老龄、仲裁事业得到加强。

（八）进一步改善人民生活，促进社会和谐稳定。实施积极的就业政策，出台了收费减免等７项配套措施，涉及资金１．２６亿元、惠及９．２１万人，全年新增就业岗位６．７５万个。创新就业载体，以百帮创业园、百帮就业一条街、街道劳务型服务公司等为依托，开展创业扶持工程。孵化企业２４７个，帮扶就业１．４万人。创建“充分就业社区”，通过组织起来就业和公益性岗位聘用，促进３．１５万下岗失业人员再就业，其中“４０５０”人员再就业率达到９８％。对２８００多个“零就业家庭”实施就业援助，实现帮扶就业率１００％。不断完善社保政策体系，保险覆盖面继续扩大，各险种参保人员较上年净增１９万人次。进一步完善相关政策使社保进入良性循环。被征地农民基本生活保障得到加强，企业退休人员社会化管理服务逐步规范，社区管理服务率达８９％。全面推进劳动合同制度，切实维护农民工合法权益，清理拖欠农民工工资２５３０万元。提高低收入者收入水平，调增最低工资标准，提高企业退休人员养老金、城镇最低生活保障、失业保险金和农村五保户补助等各类标准，惠及群众近４０万人。认真解决低收入家庭住房困难，提高廉租住房租金补贴标准，健全住房保障体系。开展“送温暖、献爱心，慈善一日捐”活动，城乡社会救助体系建设稳步推进。自觉接受人大法律监督、政协民主监督，广泛听取各方面意见和建议。办理各级人大代表议案９件、建议１７１件，办理各级政协委员提案５０２件，办结率１００％，满意度进一步提高。荣获“全国四五普法先进城市”，启动“五五”普法宣传教育，全社会法律意识进一步增强。扎实做好社会治安综合治理基层基础工作，严厉打击各种犯罪活动，８类刑事案件同比下降１９％，“平安合肥”创建工作取得突出成效。继续开展领导干部开门接访、带案下访，畅通群众诉求渠道，群体性上访事件同比下降２９％，信访秩序明显好转。创新行政复议工作方式，建立信访与复议联动机制，有效化解了行政争议。加强政府应急机制建设，应急体系建设取得重大进展。深入开展食品药品、畜产品质量安全和打击非法行医等专项整治活动，整顿规范市场秩序。健全安全生产责任制，创新监管方式，挂牌整治各类安全隐患，安全生产形势稳定。

各位代表！回顾过去一年的工作，在肯定成绩的同时，我们清醒地看到，经济社会发展中还存在一些突出矛盾和问题。如经济总量不大，辐射带动能力不强，与建设省会经济圈的要求还有差距；城乡统筹发展任务艰巨，县域经济仍然比较薄弱；制约发展的体制性障碍仍然存在，改革还需要不断深化；政府自身建设有待加强，发展环境还存在不少差距；就业和社会保障的压力较大，部分群众生产生活存在困难，等等。对这些矛盾和问题我们将高度重视，采取切实措施，认真加以解决。

二、２００７年政府工作的主要目标任务

２００７年是本届政府任期的最后一年，也是推进合肥跨越式发展、建设现代化滨湖大城市的关键一年。当前，我们正面临着十分难得的发展机遇。海外及沿海产业资本加速向内地转移，中央加强和改善宏观调控，进一步强化促进中部崛起的政策，这些都有利于我们充分发挥科教、产业、交通、区位等优势，迅速做大做强经济总量。省委、省政府对合肥发展寄予厚望，支持力度不断加大，特别是省八次党代会明确要求合肥加快现代化滨湖大城市建设，强化产业支撑，构建省会交通枢纽，提高经济首位度，形成具有较强辐射带动力的省会经济圈，为我们把握大局、加快发展进一步指明了方向。同时，全市上下已形成聚焦发展、争先进位、奋力崛起的昂扬之情，协同“三大推进”的良好大开局，成为我们乘势而上的强大动力。

按照省八次党代会的战略部署和市九次党代会及九届二次全委会提出的目标任务，今年政府工作的总体要求是：以邓小平理论和“三个代表”重要思想为指导，全面落实科学发展观，着力推进和谐社会建设，突出改革创新，坚持“三大推进”，促进又好又快发展，全力推动合肥发展再上新台阶。今年全市经济社会发展主要预期目标是：地区生产总值增长１５％以上；财政收入增长１５％；全社会固定资产投资突破１０００亿元；社会消费品零售总额增长１５％；城镇居民人均可支配收入增长１０％，农民人均纯收入增长１０％；城镇登记失业率控制在４．３％，人口出生率控制在１２‰；单位生产总值能耗降低４％左右，主要污染物排放降低２％。

为实现上述目标，今年重点做好以下１０个方面的工作：

（一）以科技创新为先导，大力推进新型工业化。以“主攻体制创新、服务知识创新、探索区域创新、突破企业创新”为重点，继续大力推进国家科技创新型试点市建设。建立以企业为主体的市、县区与校、院（所）对接体系、责任体系和服务体系，扩大合作领域，深化合作机制。充分发挥科技创新基金、风险投资基金引导作用，推进产学研合作，全力实施科技创新型企业培育计划，促进科技成果、资本和市场的对接，不断提升企业以自主品牌为核心的综合竞争力，帮助与鼓励高新技术企业迅速做大做强，全年新增高新企业５０家。加强创新人才队伍建设，完善人才激励机制，培育企业家和技术专家带头人队伍。按照总体规划、分步实施、突出重点、强化服务的原则，加快建设科技创新公共服务平台。积极创建国家知识产权示范城市。大力发展创业孵化、信息咨询等科技中介服务机构。加快推进试点市示范区建设，尽快形成产业集群创新力和区域经济创新力。继续加强各类企业技术中心、工程技术研究中心建设，支持大企业、大集团申报国家级企业技术中心。大力引进各类风险投资机构，继续举办第七届中国•合肥高新技术项目－资本对接会。积极推进城市信息化试点工作，实施“城市一卡通”、数字城管、市民信箱等项目建设，扩大信息技术的应用领域，提升城市管理和社会服务信息化水平。组织实施《全民科学素质行动计划纲要》，加快建设现代科技馆等科普设施，完善科普教育体系，提高公众科学文化素质，增强城市创新文化氛围。

全面落实“十一五”工业发展规划，开展“项目推进年”活动，保持工业发展的强劲势头。稳定和强化各项支持政策措施，扩大专项资金规模，促进工业有效投入。强力推进“１２１”工程，实施格力电器产业基地、江汽轿车、化工企业搬迁等一批重大项目建设，规划实施千亿产业和百亿元企业，促进８大产业迅速发展。围绕产业发展，建立配套产业园区及基金，促进产业集聚，提高重点产业竞争力。实施“小巨人”工程，选择一批市场前景好、发展潜力大的企业，加大政策支持和服务力度，确保２０１０年销售收入１０亿元企业达到５０户。实施“小企业振兴发展计划”，加强信用担保、发展空间等方面的服务，努力增加企业数量并使之尽快成长为规模以上企业。加快高新技术产业和节能、环保、新能源等新兴产业发展，推进再生资源回收利用体系建设，积极发展循环经济。加快发展住宅产业及建筑业，扶持建筑企业做大做强。加强工业经济运行调度监控，协调煤电油运等生产要素供给，为工业发展创造良好环境。围绕培育新的经济增长点，切实抓好项目和投资工作，努力增加投入总量，优化投资结构。

（二）深入推进县域突破战略，加快社会主义新农村建设。加大城乡统筹发展力度，坚持以城带乡、以工哺农，加快城乡规划、产业、市场和基础设施等的对接融合。以工业化为先导，迅速壮大县域工业规模。主攻先进制造业、配套工业、农副产品加工业，培育企业集聚、产业集群，提高县域工业经济比重。加速农业产业化进程，力争３县新增２－３家销售收入超１０亿元的龙头企业。构建农业产业新体系，集中建设一批种植、养殖园区，重点支持以“一村一品”、“一乡一业”为主的设施农业；实施养殖业“百千万”基地建设工程，申报国家级“无规定动物疫病区”建设项目；巩固发展林果业，加快建成全国重要的绿化苗木生产基地和肥西国家级苗木花卉市场；建立完善农业标准化生产体系，加强农产品质量安全检测监控。围绕山、水、园、林、路、庄、节做文章，充分拓展农业的休闲观光功能。加快发展农村专业技术等合作经济组织，提高农民组织化程度。继续实施劳动力转移培训“阳光工程”，提高劳务输出组织化、技能化水平。加大投入力度，推进农村基础设施建设。实施丰乐河和瓦埠湖综合治理、灌区配套与节水改造、农村安全饮水等５大工程；推进“村村通水泥路”工程建设，升级改造县乡道路，８０％以上行政村达到“村村通水泥路”目标。加快新农村“十镇百村”示范工程建设，启动实施以“四清四改”为主的村庄环境整治行动计划，改善农村居住环境。加强农作物秸秆、沼气等能源开发利用，加大农地开发整理和旧村庄整理力度，切实保护基本农田，推进农村土地集约化利用。

（三）以体制机制改革创新为重点，进一步增强发展活力与后劲。按照一切有利于发展的原则，敢闯敢试，努力把合肥建设成为中部地区乃至全国充满创新活力的“先行城市”。深入开展“改革创新年”活动，着力消除制约发展的体制机制性障碍。完成政府机构改革，深化国有资产管理体制改革，落实监管责任，强化绩效考核，提高国有资本运营质量。深化国有、集体企业改革，对列入改革计划企业，分类排队，按期推进。全面完成市属企业生活区移交辖区管理和主辅分离、辅业改制工作，妥善解决改制企业离休、协保和特殊群体人员的管理问题。深化事业单位人事制度改革，加快市政、城管、园林、供水、燃气、公交等公用事业改革，积极开展管养分离改革试点，逐步实现政府购买服务、养事不养人的目标。加快制定文化产业发展规划和扶持政策，启动文化体制改革，大力培育市场主体，整合文化资源，改革文化事业投入方式，激发文化产业发展活力。深化投融资体制改革，规范政府投资，扩大融资能力，力求投资主体多元化和城市资源增值最大化。完善重点工程建设管理体制，强化政府投资项目建设过程的组织、管理和监督工作。把发展民营经济放在突出位置，积极营造“想创业、敢创业、会创业”的社会氛围，兴起全民创业热潮。进一步强化扶持政策，扩大民营经济投资领域，吸引外地民营大企业来肥发展，扶持本土创业的民营企业，加速培育大型民营企业集团。继续深化农村综合改革，重点推进乡镇机构、农村义务教育管理、农村金融和征地制度改革，全面改善农村经济社会发展的政策环境。

（四）继续加快推进大建设，全力打造现代化滨湖大城市。围绕建设省内具有较强辐射带动力的核心增长区域，从规划入手，加强产业、政策等与周边地区的对接联合，积极打造省会经济圈。提升省会交通枢纽能力，构建省会经济圈交通框架，加快建成全国性现代物流中心。全力支持合宁、合武快速铁路建设，开工建设新机场、合肥集装箱新港，全面建成合六叶、合淮阜高速合肥段，实现环城高速全线贯通。加快滨湖新区建设，坚持“高效的基础设施先行、优质的社会事业先行”，高标准建设方兴大道、新区路网，积极推进学校、医院、商业、文化等配套设施建设，启动滨湖新区生态建设及南部水系沟通工程。大力推进主城区至４大组团及滨湖新区快速通道建设，徽州大道、蒙城北路建成通车，加快实施合安路、合马路等城市出入口道路的改扩建工程。进一步完善主城区骨干路网，全面贯通中环线，完成二环路改造，启动一环路综合畅通工程。在全面完成已开工建设道路的同时，加快黄山路、怀宁路等道路建设，完善与３大开发区的路网沟通工程。加快老城区改造步伐，重点实施三孝口、华侨饭店、安纺等区域的危旧房及城中村改造。继续加快城市公用事业建设，完成新桥水厂建设和五水厂扩建，开工建设六水厂，动工建设城市集中供热调度中心，建成一批公交保养场、换乘设施，推进城乡公交客运一体化。积极创建国家生态园林城市，开工建设野生动物园三期、高压走廊二期及二十埠河生态廊道，新建望湖城、新安江等一批城市公园。继续开展创建国家环保模范城活动，抓好全国第一次污染源调查工作。完成蔡田铺污水处理一期工程，启动十五里河和望塘污水处理二期工程，推进四里河、板桥河和二十埠河综合整治，改造老城区排水系统，实施南淝河上游污水截流和小区域污水治理工程。加快城市供电、电信、广播电视等公用事业建设。进一步提升城市管理水平，完善长效运行机制。深入开展环境综合整治，重点查处环境污染、生态破坏行为，切实加强危险废物监管。持续深入开展整治“四乱”活动，着力抓好摊群点管理、交通秩序、户外广告、城市出入口、城中村等影响城市形象的突出问题。认真落实《关于查处违法建设长效管理办法的暂行规定》，防止违法建设反弹。全面开展闲置土地清理工作，构建土地供应监督考核体系。

（五）加快发展现代服务业，提升省会综合服务功能。着眼于增强辐射带动能力，满足日益增长的需求，充分发挥省会优势，加速提升现代服务业水平。结合老城区改造和新区开发，加快中央商务区、专业批发市场、社区商业、特色商业街建设。大力发展连锁经营、电子商务等新型业态，积极培育和引进大型物流企业，实施现代物流园区等一批重大物流设施建设，推进城市商业布局调整和结构升级。继续实施“万村千乡”市场工程，建设“新网工程”，逐步提高农村连锁商业、物流配送网络覆盖面。进一步完善市级粮食储备，确保粮食安全。围绕建设区域性旅游中心，加大旅游产品开发和旅游设施建设力度，推动环巢湖旅游开发，发展乡村旅游，举办特色旅游节庆活动。壮大会展经济，抓好星级酒店、餐饮服务、会展场馆等设施建设，积极争办全国性大型会展，大力培育有潜力的区域性会展，鼓励支持会展企业做大做强。加快发展文化休闲娱乐、体育健康产业等生活服务业，培育消费热点，提升消费档次。继续推动广告、典当、拍卖、租赁等行业快速健康发展。

（六）进一步扩大对外开放，提高招商引资质量和水平。强化大开放理念，坚持面向世界、融入长三角、服务全省，积极参与中部地区分工协作，广开对外交往渠道，在更大范围、更广领域、更高层次上扩大开放，提高城市知名度和国际化程度。继续深入推进大招商，积极承接国外和沿海产业资本转移。健全目标责任体系，完善考核激励办法，强化全民招商氛围，努力实现招商引资质量、规模、领域的新突破。积极发展市场化、专业化招商，充分发挥小分队驻点招商作用，增强各类境内外招商活动的针对性、有效性。在继续抓好以８大产业为核心的制造业对外招商的同时，重点抓好本地企业增资扩股服务工作，积极鼓励和帮助本地企业做大做强。加快现代服务业、城建、农业等领域的对外开放。加强与国内外知名企业战略合作，鼓励来肥设立总部、结算中心、研发中心及销售中心，布局重大产业项目。加强与重点国家、地区华侨华人社团、企业界的联系与交往，扩大国际友城覆盖面。坚持实施科技兴贸战略，引进先进技术、设备和原辅材料，调整并优化进出口贸易结构。积极申报建设汽车及零部件出口基地城市、国际服务外包基地城市、科技创新出口基地和保税物流中心，扩大高新技术产品和机电产品出口，努力提高出口商品的国际竞争力。引导有条件的企业“走出去”开拓和扩大国际市场。进一步强化开发园区建设，突出产业分类指导和资源整合，提高投入产出率，打造园区产业特色，增强承载力和集聚辐射功能。经济区要做大做强支柱产业，加快引进“高、大、新”项目，力争新增百亿工业园１－２家。高新区要推进一批重大项目基地和产业园建设，努力成为科技创新型试点市建设的先导区。新站区要继续围绕建设商贸物流中心区的目标，进一步提升站前形象，加速站北开发。各县区工业园区要加快基础设施规划建设，健全园区服务体系，完善综合投资环境，带动区域经济快速发展。

（七）切实加强财税工作，完善金融服务体系。依法加强税收征管，确保财政收入稳定增长，实现税源扩大、收入增长与经济发展的良性循环。优化支出结构，加大重点支出保障力度，坚决压缩一般性支出。整合新农村建设资金和农业产业化发展专项资金，创新扶持工业发展方式，制定鼓励服务业发展的财税政策。加强社会保险基金使用管理，加大科技、卫生、教育和文化等公共事业投入。继续深化部门预算、国库集中支付、政府采购等改革，完善政府非税收入管理，规范市直机关津补贴发放，促进收入公平分配。加强审计监督，严肃财经纪律，提高财政资金使用效益。围绕建设区域性金融中心，进一步优化金融环境，吸引各类金融机构来肥设立分支机构和服务网点。广泛加强与世行、亚行、开行、各商业银行及其他金融机构的合作，促进资本、项目对接，拓宽融资渠道。以兴泰控股公司为抓手，发挥综合经营优势，稳步推进科技农村商业银行发展，加强地方金融服务体系建设。建立健全金融稳定协调机制，防范金融风险。以业绩为导向，整合信用担保机构，壮大担保能力，引导金融机构增加中小企业信贷，缓解中小企业融资难问题。加大企业上市力度，扩大直接融资规模。积极培育上市后备企业，支持优质企业在境内外上市、发行债券，推动上市公司再融资，全年争取３－４户企业成功上市。

（八）弘扬社会主义精神文明，大力发展各项社会事业。深入开展社会主义荣辱观学习实践活动，树立文明新风尚。积极开展学习型城市建设，促进人的全面发展。广泛深入开展文明社区、文明村镇和文明行业等群众性精神文明创建活动，确保进入全省首批文明城市行列，积极申报全国文明城市。进一步优化和调整教育布局，落实市区中小学新一轮布点规划，落实住宅小区学校同步配套建设工作，确保３３所小区配套学校竣工使用；继续推进农村中小学布局调整，加强乡镇中心学校建设，进一步改善农村中小学办学条件；全部免除城乡义务教育阶段杂费，继续实施困难家庭学生书本、住宿费补助政策；推进管理体制改革，实行校长任期责任制，加大市区校长、教师交流力度，促进教育均衡发展；扩大优质教育资源，确保滨湖高、初中新学年正式开学，创建一批示范性普高、示范性综合高中和特色高中；大力发展职业教育，统筹规划建设职业教育基地，努力打造中部地区一流的职业教育培训基地。积极发展高等教育，支持合肥学院加快发展。繁荣发展文化事业，培育文化品牌，打造城市文化特色。启动建设渡江战役纪念馆，加快建设文化艺术中心、广电中心，推进数字电视工程。加快构建公共卫生服务体系，加强应急指挥中心和疾控中心实验室建设，提高突发公共卫生事件应急处理能力；完善新型农村合作医疗制度，扩大覆盖面；加强社区卫生内涵建设，完善惠民医疗工程，提高服务水平。认真贯彻中央《关于全面加强人口与计生工作统筹解决人口问题的决定》，继续稳定低生育水平，综合治理出生人口性别比偏高等问题，完善农村部分计划生育家庭奖励扶助政策。启动第四届全国体育大会的筹备工作，参赛第六届全国城市运动会，广泛开展群众体育工作。进一步加强国防后备力量建设，提高民兵预备役建设质量，做好人防工作，争创全国双拥模范城“六连冠”。全面完成农业普查登记和数据处理，精心做好《合肥市志》续修编纂工作，全面加强震灾综合防御能力建设。重视民族宗教工作，加快民族乡（村）经济社会发展，依法管理宗教事务。认真做好外事、侨务和对台工作，大力支持工会、共青团和妇联工作，积极发展老龄人、残疾人、妇女儿童、档案、保密、气象、仲裁等事业。

（九）高度关注人民生活，努力构建和谐合肥。以落实《关于构建社会主义和谐社会若干重大问题的决定》为总纲，以解决关系人民群众切身利益问题为着力点，切实推进和谐社会建设。积极促进就业扩大再就业，新增就业岗位６万个。推动创建“充分就业社区”活动，重点开展就业困难群体就业援助工作。继续做好“创业扶持工程”，大力发展百帮创业服务中心。积极推进素质就业，全面加强高技能人才培训、创业培训、再就业培训和农村劳动力技能培训工作，提高就业竞争力。探索促进就业长效机制，尽快建立起创业培训、信用社区和小额担保贷款联动机制。完善社会保障体系，调整完善医疗保险政策，修改完善医疗保险基金结算办法；着手建立城镇未参保居民医疗保险制度，逐步提高城乡医疗救助水平；落实好机关事业单位养老保险政策，争取实现各类事业单位的全覆盖；继续强化社会保险扩面征缴，重点是非公有制企业和事业单位、高风险行业、餐饮服务行业，以及灵活就业人员、进城务工农民，保障劳动者社会保险的各项权益。开展社会化管理服务示范社区创建活动，全面提升社会化管理服务水平。落实农民工工资保障金制度，保护农民工合法权益。健全城乡社会救助体系，扩大法律援助覆盖面，建立街道、乡镇统一救助平台，全面建立农村低保制度，进一步提高农村“五保户”供养标准。加强民政基础设施建设，开工建设市社会福利中心和市新殡仪馆，规划立项救灾减灾中心和流浪未成年人救助保护中心。加强社区建设，健全新型社区管理和服务体制，开展和谐社区创建活动。继续开展“平安合肥”创建工作，完善社会治安防控体系。高度重视信访工作，坚持各级领导信访接待制度，规范信访秩序，解决合理诉求，维护群众合法权益。加强行政复议能力建设，预防和减少行政争议。完善公共安全应急体系，提高突发事件和社会风险的预警应急能力。深入开展严打整治和矛盾纠纷化解工作，维护社会政治稳定。推进“三基”工程和全国科技强警示范城市建设，建成特警基地。加强消防安全工作，加快消防设施建设。继续推进食品药品放心工程，打击非法行医。严格落实安全生产责任制，健全监管、救援、监督体系，切实加强安全生产工作。

（十）加强政府自身建设，推进政府管理创新。进一步履行好政府职能。创新经济管理制度和方式，更多地运用经济手段和法律手段调节经济活动。强化市场监管，深入整顿规范市场秩序，严厉打击扰乱市场的违法行为。创新公共服务体制，改进公共服务方式，把政府工作的重点、政策支持的重点、财力保障的重点进一步向社会事业倾斜，向公共基础设施建设倾斜，向农村、基层、薄弱地区和城市困难群体倾斜，向生态建设、环境保护倾斜，向扩大就业、完善社会保障体系倾斜。进一步提高依法行政水平。贯彻落实国务院依法行政纲要，强化行政执法监督，规范行政执法行为。加强电子政务和信息化建设，扩大政务公开渠道和范围，建成并有效运行并联审批系统。坚持科学民主决策，健全重大事项集体决策、专家咨询、社会公示与听证、决策评估等制度。建立健全决策反馈纠偏机制和决策责任追究制度。自觉接受人大法律监督和政协民主监督，认真办理人大议案、代表建议和政协委员提案，加强与各民主党派、工商联的沟通与联系，高度重视和支持人民群众与新闻舆论监督。进一步加强效能建设。围绕尽快把合肥打造成中西部地区乃至全国审批环节最少、办事效率最高的城市之一，加强各级行政服务中心建设，形成上下联动工作机制，提高整体服务水平。进一步创新体制和机制，减少和规范行政审批事项，对已取消的审批项目，切实加强后续监管，坚决杜绝各种变相审批行为。理顺部门职能分工，优化工作流程，做到运转更加规范协调、工作更加高效便民。建立健全效能建设长效机制，逐步形成与政府管理创新相适应的激励约束机制和社会评价机制，探索建立科学统一的政府绩效考核指标体系。进一步加强勤政廉政建设。全面加强新形势下的领导干部作风建设。各级领导干部要大力弘扬求真务实的作风，深入基层，深入群众，多办顺应民意、化解民忧、为民谋利的实事；要秉公用权、廉洁从政，自觉遵守国家的法律法规，严格执行领导干部廉洁从政的各项规定。建立健全教育、制度、监督并重的惩治和预防腐败体系，从源头上铲除腐败滋生蔓延的土壤。加强行政监察、审计监督、财政监督和政府督查工作，坚决惩治腐败行为和不正之风。倡导勤俭节约之风，反对铺张浪费。坚持从严治政，不断提高公务员队伍政治素质、业务素质和办事能力，以过硬的素质、优良的作风和奋发有为的精神状态，履行好党和人民赋予的神圣职责，努力建设高效、务实、廉洁、亲民的政府。

各位代表，我们正处在一个改革创新的时代。我们已经绘就并开始实施我们的宏伟蓝图。让我们在省委、省政府和市委的坚强领导下，以邓小平理论和“三个代表”重要思想为指导，以科学发展观统揽各项工作，解放思想，开拓创新，勇当先锋，为加快推进现代化滨湖大城市建设，早日实现全面建设小康社会目标而努力奋斗。
